# Diagnostic Pathways of *Leptospira* spp. in Dogs with Kidney Injury

**DOI:** 10.3390/pathogens13090792

**Published:** 2024-09-12

**Authors:** Ioan Hutu, Oana Maria Boldura, Iasmina Luca, Sorin Aurelian Pasca, Alina Andreea Dragoescu, Radu Valentin Gros, Bianca Cornelia Lungu, Andrei Călugăriță, Cornel Baltă, Călin Mircu, Adrian Constantin Stancu

**Affiliations:** 1 *Horia Cernescu* Research Unit, Faculty of Veterinary Medicine, University of Life Sciences “Regele Mihai I”, 300645 Timisoara, Romania; ioan.hutu@fmvt.ro (I.H.); oanaboldura@usvt.ro (O.M.B.); valentingros@usvt.ro (R.V.G.); bianca.lungu@fmvt.ro (B.C.L.); andrei.calugarita@fmvt.ro (A.C.); adrianstancu@usvt.ro (A.C.S.); 2 Faculty of Veterinary Medicine, University of Life Sciences, 700490 Iasi, Romania; sorin.pasca@iuls.ro; 3 Faculty of Agriculture, University of Life Sciences “Regele Mihai I”, 300645 Timisoara, Romania; andreeadragoescu@usvt.ro; 4 Aurel Ardelean Institute of Life Sciences, Vasile Goldis Western University of Arad, 310025 Arad, Romania; balta.cornel@uvvg.ro

**Keywords:** leptospirosis, kidney, diagnosis, molecular biology, immunohistochemistry, dogs

## Abstract

Pathogenic *Leptospira* spp. causes leptospirosis in animals and humans globally, leading to systemic infections that can impact vital organs in affected animals. The purpose of this study was to evaluate kidney injury and to perform a retrospective analysis of leptospirosis infection in follow-up dog samples. The retrospective study collected epidemiological information obtained through paraclinical exams, immunohistochemistry (IHC), and molecular biology (qPCR) of cases from the Faculty of Veterinary Medicine from Timisoara between September 2016 and May 2023. No correlations were found between *Leptospira* infection and breed (*p* = 0.714), gender or castration status (*p* = 0.890), and anatomic pathology exam results (*p* = 0.608). Significant associations were found in cases with high levels of azotemia (*p* = 0.000) and immunological status (vaccinated vs. unvaccinated, *p* = 0.000), with the leptospirosis risk in unvaccinated animals calculated at OR = 85.500 (95%CI, 6.82–1071.26, *p* = 0.000). Retrospectively, leptospirosis was diagnosed in 27/65 cases (42%) using the IHC method, while the qPCR assay detected 29/65 cases (45%). This study demonstrates that qPCR is a robust and specific method for postmortem diagnosis of *Leptospira* spp. infection in dogs, offering higher specificity and reliability compared to traditional IHC methods, which showed 94.74% specificity in our study.

## 1. Introduction

Leptospirosis, caused by bacteria of the genus *Leptospira*, is a zoonotic disease; all species of homeothermic animals and humans are receptive to leptospiric infection. The receptivity of heterothermic animals is questionable, but positive serological reactions have also been found. Of domestic mammals, the most receptive ones are pigs, bulls, and dogs. The infection pathways are multiple. Of these, the direct path seems to be the most common—humans and animals may become infected through direct contact with the urine, blood, or tissue of infected animals. Indirect transmission is also common, through contact with water, soil, or food that has been contaminated with the urine of infected animals. *Leptospira* can also penetrate through wounds or erosions of the skin and mucous membranes, sometimes even through intact skin; cases of transmission of the disease through sexual contact have also been reported. *Leptospira* transmission is facilitated by hot and humid weather, when germs find better survival conditions in the environment and when humans and dogs have increased contact with infected waters [1,2,3]. Puppies manifest clinical infection more often, because they are more susceptible, whereas subclinical infections are more frequent in adults or older dogs. Diseased animals eliminate *Leptospira* for several days through various secretions and excretions. After passing through the disease course, they eliminate *Leptospira* through the urine, thus contaminating the environment.

Clinically, leptospirosis displays a wide range of symptoms, among which acute renal failure is one of the predominant and most severe aspects. Assessing dogs for leptospirosis infection can be carried out through a variety of methods. Bacterial culture is the standard method in retrospective immunohistochemistry for *Leptospira* detection in dogs with kidney injury. Obtaining a *Leptospira* culture is technically difficult, and the administration of antibiotics may affect the growth of bacteria, which can lead to a false-negative diagnosis. The immunofluorescence assay is useful for the identification of *Leptospira*, which is commonly identified in urine and blood, as well as tissue. Because subclinical shedding has been documented in shelter dogs [4,5], leptospirosis may be more common than the number of diagnoses would suggest. Serologic examination may be used to detect subclinical cases. A safe method of identifying *Leptospira* is is immunohistochemistry [6,7,8,9,10,11,12].

A 2007 study conducted in several states across the USA found that about 17–25% of the examined dogs had no clinical signs of anti-Leptospira antibodies against one or more serotypes found in dogs. This shows that there was exposure to *Leptospira* [13,14,15,16,17]. The increased incidence of clinical leptospirosis in dogs and serological data suggests that subclinical infection is associated with chronic kidney injury [1,2,18]. Initially, the clinical manifestation of leptospirosis in dogs is kidney injury (tubulointerstitial nephritis). The progression of tubulointerstitial nephritis to renal fibrosis has been described in dogs [19,20], which becomes a cause of mortality in older dogs [21].

The identification and subsequent detection of the *lipL32* gene hold significant value in the context of epidemiological surveillance to ascertain the prevalence of pathogenic *Leptospira* spp. [22]. The *lipL32* gene exhibits high conservation among pathogenic *Leptospira* spp. [23,24]. This gene encodes a surface-exposed lipoprotein anchored to the bacterial cell membrane, and it serves a pivotal function in mediating the interactions between the bacterium and its host. Notably, this gene is exclusively present in pathogenic *Leptospira* species [25,26], being frequently employed as the focal point in polymerase chain reaction (PCR) assays devised to detect *Leptospira* DNA within clinical specimens [27]. The abundance of the *lipL32* gene in *Leptospira* bacteria makes it exceptionally useful as a target in PCR-based detection methodologies, thereby markedly augmenting the sensitivity of the diagnostic test [28]. Even minute quantities of *Leptospira* cells within clinical samples can be detected through the amplification of this gene [29].

The aim of the study is to compare immunohistochemistry (IHC) and molecular biology (qPCR) diagnosis methods in a retrospective analysis of leptospirosis infection in follow-up dog samples. The epidemiological information has been corroborated with results obtained by applying IHC and qPCR methods in dogs diagnosed with kidney injury.

## 2. Materials and Methods

### 2.1. Case Selection and Criteria for Inclusion in the Study

The study was conducted on 65 dogs with kidney injury of any type recorded between September 2016 and May 2023, both in the university clinic and in cases from private clinics from Western Romania. The cases were selected in chronological order and consisted of biopsy samples and autopsies performed between 2016 and 2023. The total number of 65 dogs included in the study belonged to several breeds, as shown in the case distribution table below (Table 1).

Retrospectively, as a routine practice, the clinical assessment focused on the patient’s history (including vaccination status) and clinical signs. Laboratory tests that were commonly performed included a complete blood count (looking for leukocytosis, thrombocytopenia, and anemia), serum biochemistry (evaluating liver enzymes such as ALT, AST, and ALP, azotemia indicated by elevated creatinine, hyperbilirubinemia, and electrolyte imbalances), urinalysis (assessing urine specific gravity and sediment), and serological tests (measuring antibody titers for leptospirosis).

Following the postmortem examinations performed, inflammatory processes, degenerative processes, and neoplasms were found and grouped into four categories: category 1—glomerulonephritis as a predominant lesion, category 2—chronic interstitial nephritis, category 3—acute interstitial nephritis, and 4—other lesions (including neoplasia, amyloidosis, congestion, bleeding, etc.).

### 2.2. Post Mortem Detection of Leptospirosis by Immunohistochemical Examination

In performing the IHC, several steps were performed:

Sample Preparation: No IHC examinations were performed on samples from the dogs that had received antibiotic therapy. The tissue samples were fixed in formalin and embedded in paraffin. The paraffin block was sectioned in 4–5 μm layers and immersed in a distilled water bath at 40 °C. The obtained sections were laid on glass slides and dried before immunostaining.

Tissue sections were deparaffinized using xylene and rehydrated by successive immersion in ethanol solutions of decreasing concentrations for five minutes (from 100 to 95, 70, and 50%).

Antigen requires blocking endogenous peroxidase activity by incubating the sample in a 3% hydrogen peroxide–methanol solution for ten minutes at 24 °C and washing with phosphate-buffered saline (PBS). Then, the antigen that was made with citrate buffer (pH 6.0) was incubated, according to the manufacturer’s instructions.

Blocking non-specific binding with bovine serum albumin: 10% PBS was used to prevent the non-specific binding of antibodies. Tissue sections were incubated with the primary antibody (*Leptospira interrogans,* Dilution Ratio: 1/50 [30]), specific for *Leptospira* antigens and incubated in a humid chamber at 24 °C for one hour.

Secondary antibody incubation (Dilution Ratio: 1/500 [31]): After it was washed off twice, the primary antibody was incubated for 30 min with diluted biotinylated secondary antibodies. The secondary antibody binds to the primary antibody.

*Detection:* The secondary antibodies were nonbiotinylated, and then the enzyme attached to avidin was incubated with the substrate to generate a precipitate visualized under optical microscopy.

Counterstaining and mounting: Tissue sections were counterstained with hematoxylin to highlight cellular structures. The slides were mounted with a cover slip for examination under a microscope for the interpretation and quantification of antigen expression in the investigated tissue.

### 2.3. Post Mortem Confirmation of Leptospirosis by PCR

To confirm the clinical evaluation results, the SYBR Green qPCR assay was employed as suggested by Sripattanakul et al., 2022 [27]. It targeted *lipL32*, a gene found only in pathogenic *Leptospira* spp., including *L. canicola*. All samples were analyzed in triplicate by PCR amplification of the *lipL32* gene (used for the detection of pathogenic *Leptospira* strains), along with a negative template control. Total genomic DNA was isolated in 65 biological samples from 50 mg of kidney tissue by using the NucleoSpin^®^ DNA Clean-Up XS kit (Machery-Nagel, Düren, Germany), according to the manufacturer’s protocol. The quality and quantity of isolated DNA were assessed by the spectrophotometric method using a NanoDrop8000 Instrument (Thermo Fisher Scientific, Waltham, MA, USA). An amount of 100 ng of DNA was used as a template in PCR reactions and amplified using the primers described in the literature [27]: Lep F GGCGGCGCGTCTTAAACATG and Lep R TCCCCCCATTGAGCAAGATT, using the GoTaq^®^ qPCR Master Mix (Promega, Madison, WI, USA) with an ABI 7500 Real Time PCR System (Applied Biosystems, Waltham, MA, USA) in an experiment of present/absent type. The polymerase was activated at 95 °C for 2 min, followed by 45 cycles of denaturation at 95 °C for 15 s and annealing/extension at 60 °C. The melting curve was analyzed at 60–95 °C, 0.5 °C increments at 5 s/step. A cycle threshold (Ct) under the value of 38 was considered a positive result for pathogenic *Leptospira* spp.

### 2.4. Statistical Analysis

The statistical test used for interval or continuous variables was the Kruskal–Wallis test (non-parametric). Frequencies were analyzed using Pearson’s chi-squared test. These statistical analyses were performed using SPSS Statistics for Windows, Version 17.0 (SPSS Inc., Chicago, IL, USA). Statistical significance was considered at *p* values of <0.05.

## 3. Results

### 3.1. Clinical and Epidemiological Outcome

The average age of the dogs was 7.28 ± 0.40 years. The study found no correlation between the age of the dogs and their reaction to *Leptospira* infection (t = 0.131, *p* = 0.896), according to the IHR test. Additionally, the study found no significant association between breed and *Leptospira* infection (χ^2^ = 2.908, *p* = 0.714, Table 1) in dogs that tested positive in the IHC exam. Gender was neither correlated with positive reactions to *Leptospira* (χ^2^ = 0.074, *p* = 0.786, data from Table 2) nor with the reproductive status of the dogs (χ^2^ = 0.019, *p* = 0.890, data from Table 2).

The classes related to the immunological status of the dogs from Table 3 appear to be associated with a positive IHC reaction to *Leptospira* infection (χ^2^ = 18.961, *p* = 0.000). Leptospirosis and the vaccinated vs. unvaccinated status (Table 3) were significantly correlated (χ^2^ = 19.164, *p* = 0.000). The risk of leptospirosis in the unvaccinated animals in our study was calculated at OR = 85.500 (95% CI, 6.82–1071.26, at *p* = 0.000). From the statistics presented in Table 3, it appears that unvaccinated dogs are at a higher risk of leptospirosis, according to the ICH exam.

### 3.2. Paraclinical Exams

Typically, regarding the antibody status, a result was considered positive if the values were >1:800. Using the International Renal Interest Society (IRIS) staging system to categorize (as detailed in Table 4) the severity of kidney disease [19] based on elevated creatinine levels, the relevant biochemistry exams revealed the following in 65 dogs: 6 dogs exhibited severe renal azotemia (9.58 ± 0.75 mg/dL), 8 dogs had moderate renal azotemia (3.49 ± 0.29 mg/dL), 7 dogs showed mild renal azotemia (1.70 ± 0.08 mg/dL), and 25 dogs were diagnosed with non-azotemic kidney disease (0.88 ± 0.06 mg/dL). The differences were significant according to the IRIS classification (Kruskal–Wallis test,χ^2^ = 37.317, *p* = 0.000). In terms of frequencies, based on immunohistochemistry, azotemia appeared to be associated with the presence of the leptospiral antigen (χ^2^ = 23.846, *p* = 0.000, Table 4).

### 3.3. Histopathological Examination

As shown by the histopathological examination, 8/65 of dogs (12%—Table 5) had acute interstitial nephritis (inflammatory infiltrate in the kidney interstitium), 14/56 (22%) had chronic interstitial nephritis (chronic inflammation in the renal interstitium), and 37/65 (57%) had glomerulonephritis (inflammation and damage to the glomerulus).

Of these, *Leptospira* was found in 63%, 43%, and 38% at the IHC exam, but the statistical values do not correlate with the classes of renal pathology (χ^2^ = 1.833, *p* = 0.608, Table 5). Other lesions such as neoplasia, amyloidosis, congestion, bleeding, etc., were also identified.

### 3.4. Immunohistochemical Examination

**Figure 1:**
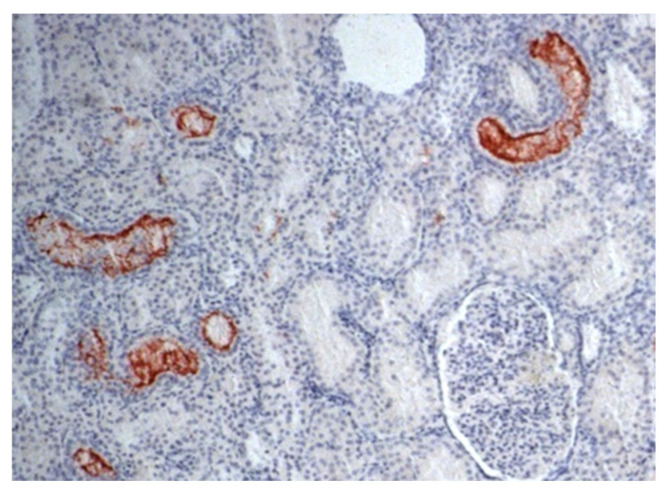
Photomicrography of renal parenchyma in a dog. IHC for leptospirosis kidney: *Leptospira* immunohistochemistry showed positive immunoprecipitation of *Leptospira* antigens in the cytoplasm of tubular cells—brownish aggregates that adhered to tubular epithelial cells and projected into the lumen (400× magnification).

**Figure 2:**
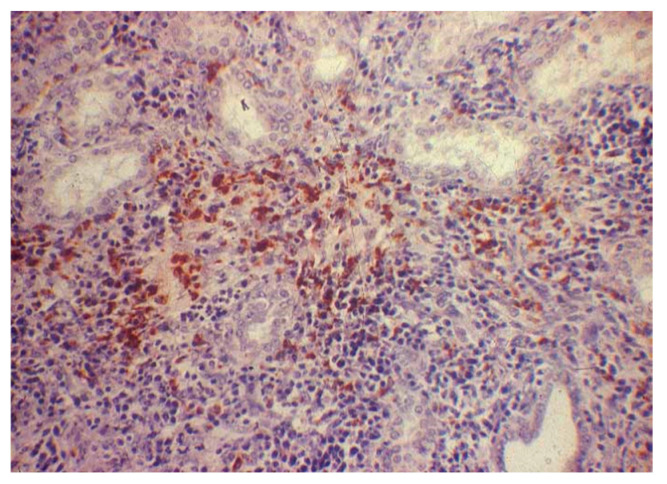
Canine kidney. IHC for leptospirosis kidney. H&E staining showing interstitial nephritis with scarce inflammatory infiltrate mainly composed of mononuclear cells (200× magnification).

Overall, the results of the study showed that 42% (27/65) of dogs had renal pathology associated with *Leptospira*, according to the immunohistochemistry (IHC) method (Figure 1 and Figure 2 and Table 1, Table 2, Table 3, Table 4 and Table 5).

The positive cases in our study could not be associated with variables such as breed (Table 1), gender, and sterilization procedure (Table 2), or anatomopathological kidneypathology (Table 4). However, the disease, as confirmed by the IHC exam, was associated with azotemia levels (Table 4) and with leptospiral immunological status (Table 3).

### 3.5. Diagnostic Confirmation by qPCR Analyses

A total of 65 canine samples were subjected to qPCR analysis to confirm postmortem diagnoses of *Leptospira* infection initially identified through immunohistochemistry (IHC) examinations. Out of these, the 29 samples that were found positive for *Leptospira* spp. in the IHC also yielded positive results in the qPCR analysis, with cycle threshold values consistently below 36. Also, two other samples that were not confirmed by IHC were considered positive, since the Ct values were under 38–36.4762 and 37.5219, respectively (Table 6).

In detail, 16 of the 29 positive samples had Ct values below 27, indicating a more advanced stage of infection. The remaining 13 positive samples exhibited Ct values ranging from 27 to 37.5219, suggesting either a lower bacterial load in the kidney tissue collected or a less severe infection. Negative controls (NTCs) showed no amplification, confirming the accuracy of the qPCR method. Furthermore, melting curve analysis revealed no non-specific product formation or primer-dimer artefacts, underscoring the specificity and reliability of the DNA-based diagnostic approach. These results corroborate the initial IHC findings and demonstrate the high accuracy and reliability of qPCR in detecting pathogenic *Leptospira* spp. Our study indicates that qPCR not only identifies the presence of the pathogen more precisely than IHC, but also provides an assessment of infection severity. In the case of kidney tissue samples, using the IHC method, we identified 27 positive cases and 38 negative cases; whereas, by using the qPCR method, we identified 29 positive cases and 36 negative cases. If we assume the qPCR method has a sensitivity of 100% as cited in the literature [32], the IHC method produced twofalse-negative results. Therefore, in our study, the specificity of the IHC method can be calculated and would be 94.74% [36/(2 + 36)].

## 4. Discussion

Because all available diagnostic tests have limitations, the application of a combination of serologic assays and organism detection tests is recommended to optimize diagnosis of leptospirosis [19]. In the case of follow-up kidney dog samples, immunohistochemistry and molecular biology (qPCR) diagnosis methods are recommended [1,12,33]. Sykes et al. (2023), in the American College of Veterinary Internal Medicine (ACVIM) consensus statement on leptospirosis in dogs, established clinical and laboratory criteria for confirming a case. In accordance with the consensus statement, a confirmed case must meet the clinical criteria and at least one of the following confirmatory laboratory criteria: (i) a fourfold or higher increase in *Leptospira* agglutination titer between acute-and convalescent-phase serum specimens at a single laboratory, (ii) the detection of pathogenic leptospires in blood using a nucleic acid amplification test (NAAT), or (iii) isolation of *Leptospira* from a clinical specimen by a *Leptospira* reference laboratory [19].

The presence of anti-Leptospira antibodies against one or more serotypes shows that there was exposure to *Leptospira* [13,14,15,16,17]. The results obtained were compared with the PCR test and the serological examination, which showed that in the urine tests, about 8% (5 cases out of 65) were positive, although only about 3% (2 cases out of 65) of the dogs studied displayed clinical signs [1,14,26]. A proportion of the dogs (27/65, or 42%) tested positive in the IHC, displaying a reaction to *Leptospira* infection. The positive immunohistochemical result is explained by the persistence of the leptospiric antigen for a longer time. The use of the immunohistochemical method has been shown to be effective in identifying leptospiral antigens, and it is the only technique that can be applied currently in legal veterinary diagnostic laboratories. However, serotypes in the renal tissue cannot be determined by the immunohistochemical method, because the antibodies produce cross-reactions between serotypes. Thus, serum antibody titers from infected dogs cannot provide information regarding infected leptospiral serotypes or vaccinal leptospiral fragments. All leptospiral vaccines contain whole or fragmented inactivated leptospiral organisms. Immunohistochemistry has been shown to have sensitivity and specificity for *Leptospira* similar to the silver staining of renal tissue. Some authors have suggested that the role of leptospirosis in chronic interstitial nephritis [34] is unclear, because the evolution of histopathological changes in subacute and chronic forms cannot be observed. This uncertainty is explained by the absence of antibody titers and the non-identification of leptospires in histopathological examinations. Even though IHC is a powerful diagnostic tool, its application in detecting leptospirosis in dogs can be limited by several factors. These include antigen availability [3,19], antibiotic use [35], sample quality, antibody specificity, technical demands, and individual variations in immune response [36]. These limitations must be carefully considered to interpret the IHC results accurately. For all the above-mentioned reasons, we suspect that the IHC results were perhaps influenced by undeclared antibiotic therapy.

On the other hand, rapid diagnosis of leptospirosis can be difficult without adequate expertise and it is often delayed due to the time needed to obtain results. Polymerase chain reactions (PCRs) are methods for real-time detection of amplified PCR products, which provides a better diagnosis of culture and serology [22,26,34]. Nowadays, there are multiple real-time PCR (qPCR) methods for detecting *Leptospira*, but not all of them can distinguish pathogenic and non-pathogenic species [24]. Furthermore, various probe technologies and qPCR instruments are used for these tests [19,36,37,38,39,40,41], including kidney samples [37]. Chandan 2016 [42] developed a sensitive PCR assay targeting a specific sequence from the *Leptospira canicola*, which detected as few as ten bacteria and was suitable for diagnosing leptospirosis in humans. Flores 2020 [25] presented a protocol for rapidly detecting leptospiral DNA in environmental water using a TaqMan-based qPCR targeting the *lipl32* gene, which was specific to pathogenic *Leptospira* spp. These findings collectively demonstrated the utility of RT-qPCR in detecting *Leptospira* spp.

Compared to the conventional methods, the advantages of the qPCR detection method are that it is fast, it reduces the chances of contamination, it is specific and sensitive, and it has a high through put [19,43,44,45,46]. The qPCR assay has been found to detect as low as 10^2^ and 10^3^ bacteria/mL of pure culture, whole blood, plasma, and serum samples targeting the *lipL32* gene regions [47,48]. Our study demonstrates that qPCR is a robust and specific method for postmortem diagnosis of *Leptospira* spp. infection in dogs, offering higher specificity and reliability compared to traditional IHC methods, which showed 94.74% specificity in our case. In addition, the advantages of qPCR for detecting *Leptospira* lie in its sensitivity, specificity, speed, quantitative capability, automation, versatility, early detection potential, robustness, and molecular typing capability. These features make qPCR a valuable tool in both clinical and research settings for diagnosing and studying leptospirosis.

## 5. Conclusions

Immunohistochemical examination has been shown to be effective in identifying leptospiral antigens, and it is the only technique that can be applied currently. Serotypes in the renal tissue cannot be determined by the immunohistochemical method, because the antibodies produce cross-reactions between serotypes, and results may be influenced by other factors. Consequently, the IHC and real-time PCR (qPCR) method shave the potential to increase the accuracy of *Leptospira* detection and postmortem diagnosis.

## Figures and Tables

**Table 1:** Case distribution in a retrospective study.

Breed of Dog	Number of Cases	Reaction to *Leptospira* ^1^	
		Positive	Negative
German Shepherd Dog	14 (22%)	5 (36%)	9 (64%)
Rottweiler	8 (12%)	2 (25%)	6 (75%)
Dobermann	12 (18%)	5 (42%)	7 (58%)
Labrador	17 (26%)	9 (53%)	8 (47%)
Boxer	3 (5%)	2 (67%)	1 (33%)
Mixed breed	11 (17%)	4 (33%)	7 (64%)
**Total**	**65 (100%)**	**27 (42%)**	**38 (58%)**

^1^ The positive samples in accordance with the IHC test.

**Table 2:** Case distribution in a retrospective study.

Gender and Sterilization Procedure	Number of Cases with Kidney Injury	Reaction to *Leptospira* ^1^	
		Positive	Negative
Unsterilized female	6 (9%)	2 (33%)	4 (67%)
Sterilized female	29 (45%)	12 (41%)	17 (59%)
Uncastrated male	9 (14%)	4 (44%)	5 (56%)
Castrated male	21 (32%)	9 (43%)	12 (57%)
**Total**	**65 (100%)**	**27 (42%)**	**38 (58%)**

^1^ The positive samples in accordance with the IHC test.

**Table 3:** Leptospiral immunological status.

Immunological Status ^2^	Number of Cases	Reaction to *Leptospira* ^1^	
		Positive	Negative
Vaccinated	21	2	19
Unvaccinated	10	9	1
Unknown situation	34	16	18
	**65 (100%)**	**27 (42%)**	**38 (58%)**

^1^ The positive samples in accordance with the IHC test. ^2^ The correlation between the positive results and azotemia, Pearson χ^2^ = 18.961, df = 2, *p* = 0.000.

**Table 4:** Presence of azotemia ^2^, by creatinine level.

Specification ^2^	Number of Cases	Reaction to *Leptospira* ^1^	
		Positive	Negative
Stage 4: severe renal azotemia (>5.0 mg/dL)	6 (9%)	4 (15%)	2 (5%)
Stage 3: moderate renal azotemia (2.1–5.0 mg/dL)	8 (12%)	5 (19%)	3 (8%)
Stage 2: mild renal azotemia (1.4–2.0 mg/dL)	7 (11%)	6(22%)	1 (3%)
Stage 1: non-azotemic kidney disease (<1.4 mg/dL)	25 (38%)	1 (4%)	24 (63%)
Unknown value of azotemia	19 (29%)	11 (41%)	8 (21%)
**Total**	**65 (100%)**	**27 (42%)**	**38 (58%)**

^1^ The positive samples in accordance with the IHC test. ^2^ The association between the positive results and the IRIS classes (Stage 1 to Stage 4), Pearson χ^2^ = 23.846, df = 3, *p* = 0.000.

**Table 5:** Incidence of renal pathology.

Anatomo Pathological Diagnosis	Number of Cases	Reaction to *Leptospira* ^1^	
		Positive	Negative
Acute interstitial nephritis(AIN)	8 (12%)	5 (63%)	3 (38%)
Chronic interstitial nephritis (CIN)	14 (22%)	6 (43%)	8 (57%)
Glomerulonephritis (GLN)	37 (57%)	14 (38%)	23 (62%)
Other	6 (9%)	2 (33%)	4 (67%)
**Total**	**65 (100%)**	**27 (42%)**	**38 (58%)**

^1^ The positive samples in accordance with the IHC test.

**Table 6:** qPCR results of canine samples tested for *Leptospira canicola*.

Dog Breed	Number of Cases	Reaction to *Leptospira* ^1^	
		Positive	Negative
German Shepherd Dog	14 (22%)	6 (43%)	8 (57%)
Rottweiler	8 (12%)	2 (25%)	6 (75%)
Dobermann	12 (18%)	5 (42%)	7 (58%)
Labrador	17 (26%)	10 (59%)	7 (41%)
Boxer	3 (5%)	2 (67%)	1 (33%)
Mixed breed	11 (17%)	4 (33%)	7 (64%)
**Total**	**65 (100%)**	**29 (45%)**	**36 (55%)**

^1^ The positive samples according to the qPCR test.

## Data Availability

The data presented were obtained from all subjects involved in this study.
